# Quantitative assessment of angioplasty-induced vascular inflammation with ^19^F cardiovascular magnetic resonance imaging

**DOI:** 10.1186/s12968-023-00964-7

**Published:** 2023-10-03

**Authors:** Fabian Nienhaus, Moritz Walz, Maik Rothe, Annika Jahn, Susanne Pfeiler, Lucas Busch, Manuel Stern, Christian Heiss, Lilian Vornholz, Sandra Cames, Mareike Cramer, Vera Schrauwen-Hinderling, Norbert Gerdes, Sebastian Temme, Michael Roden, Ulrich Flögel, Malte Kelm, Florian Bönner

**Affiliations:** 1 Division of Cardiology, Pulmonology and Vascular Medicine, University Hospital and Medical Faculty, Heinrich-Heine-University, Moorenstr. 5, 40225 Düsseldorf, Germany; 2 Institute for Clinical Diabetology, German Diabetes Center, Leibniz Center for Diabetes Research at Heinrich Heine University, Düsseldorf, Germany; 3 German Center for Diabetes Research, Partner Düsseldorf, Düsseldorf, Germany; 4 Central Animal Research Facility, Heinrich Heine University, Düsseldorf, Germany; 5 Experimental Cardiovascular Imaging, Department of Molecular Cardiology, Medical Faculty, Heinrich-Heine-University Düsseldorf, Düsseldorf, Germany; 6 Division of Endocrinology and Diabetology, Medical Faculty, Heinrich Heine University, Düsseldorf, Germany; 7 Cardiovascular Research Institute Düsseldorf (CARID), Medical Faculty, Heinrich-Heine-University, Düsseldorf, Germany; 8 Experimental Anesthesiology, Medical Faculty, Heinrich Heine University, Düsseldorf, Germany; 9 Department of Clinical and Experimental Medicine, University of Surrey, Faculty of Health and Medical Sciences, Guildford, UK; 10 Department of Vascular Medicine, Surrey and Sussex Healthcare NHS Trust, Redhill, UK

## Abstract

**Background:**

Macrophages play a pivotal role in vascular inflammation and predict cardiovascular complications. Fluorine-19 magnetic resonance imaging (^19^F MRI) with intravenously applied perfluorocarbon allows a background-free direct quantification of macrophage abundance in experimental vascular disease models in mice. Recently, perfluorooctyl bromide-nanoemulsion (PFOB-NE) was applied to effectively image macrophage infiltration in a pig model of myocardial infarction using clinical MRI scanners. In the present proof-of-concept approach, we aimed to non-invasively image monocyte/macrophage infiltration in response to carotid artery angioplasty in pigs using ^19^F MRI to assess early inflammatory response to mechanical injury.

**Methods:**

In eight minipigs, two different types of vascular injury were conducted: a mild injury employing balloon oversize angioplasty only (BA, n = 4) and a severe injury provoked by BA in combination with endothelial denudation (BA + ECDN, n = 4). PFOB-NE was administered intravenously three days after injury followed by ^1^H and ^19^F MRI to assess vascular inflammatory burden at day six. Vascular response to mechanical injury was validated using X-ray angiography, intravascular ultrasound and immunohistology in at least 10 segments per carotid artery.

**Results:**

Angioplasty was successfully induced in all eight pigs. Response to injury was characterized by positive remodeling with predominantly adventitial wall thickening and concomitant infiltration of monocytes/macrophages. No severe adverse reactions were observed following PFOB-NE administration. In vivo ^19^F signals were only detected in the four pigs following BA + ECDN with a robust signal-to-noise ratio (SNR) of 14.7 ± 4.8. Ex vivo analysis revealed a linear correlation of ^19^F SNR to local monocyte/macrophage cell density. Minimum detection limit of infiltrated monocytes/macrophages was estimated at approximately 410 cells/mm^2^.

**Conclusions:**

In this proof-of-concept study, ^19^F MRI enabled quantification of monocyte/macrophage infiltration after vascular injury with sufficient sensitivity. This may provide the opportunity to non-invasively monitor vascular inflammation with MRI in patients after angioplasty or even in atherosclerotic plaques.

**Supplementary Information:**

The online version contains supplementary material available at 10.1186/s12968-023-00964-7.

## Introduction

Vascular inflammation plays a central role in the progression of atherosclerosis in coronary artery disease (CAD) as well as peripheral artery disease (PAD) leading to subsequent cardiovascular complications [1]. The influx and accumulation of circulating monocytes is a key feature in the progression from stable to vulnerable atherosclerotic plaques [2] as the number of monocytes/macrophages directly correlates to plaque vulnerability [3]. Consequently, the visualization of certain vascular danger patterns in the transition from asymptomatic to symptomatic disease might ideally identify patients with critical disease progression [4]. However, in current clinical practice there is no established non-invasive imaging technique to quantitatively visualize these danger pattern, especially the accumulation of macrophages, with a clear positive contrast. ^19^F MRI might be a promising imaging technique to overcome these limitations.

We have shown that ^19^F MRI is feasible to visualize monocytes/macrophages in experimental disease models in mice at experimental field strength [5, 6]. This approach is based on the properties of monocytes/macrophages to ingest fluorine-containing perfluorocarbon-nanoemulsions (PFC-NE) that allows specific cell tracking in vivo [7]. As the ^19^F signal is directly proportional to the amount of the PFC compound and there is a negligible natural ^19^F-background in the mammalian body, this imaging technique is highly specific and directly quantifiable by its positive contrast [5]. ^19^F MRI may provide an ideal platform to visualize vascular inflammation and to quantify monocytic cell accumulation in vivo in the arterial wall [4]. Recently, ^19^F MRI demonstrated feasibility and applicability by using clinical scanners with sufficient signal-to-noise ratio (SNR) and reasonable scan time (< 20 min) to reveal local inflammatory pattern after myocardial infarction in mice [8, 9] and pigs [10–12]. Whether this approach can be used in large animal models of vascular inflammation is currently unknown. Considering the promising background of imaging vascular inflammation in mice and first translational evidence in pigs with clinical scanners, this study aimed to prove feasibility of ^19^F MRI to visualize accumulation of monocytes/macrophages in the arterial wall in a model of balloon angioplasty-induced vascular injury.

## Methods

### Model of vascular injury

The experiments were performed in eight adult Aachen minipigs [13] with an mean age of 2 years (± 5 months) a mean thorax girth of 110 ± 35 cm with a body weight of 72 ± 15 kg, which were bred, housed and extensively characterized [12] at the central animal facility center of Heinrich-Heine-University, Düsseldorf, Germany. All study protocols were carried out in accordance to the national guidelines of animal care and approved by the state authority ‘Landesamt für Natur-, Umwelt- und Verbraucherschutz’ (84-02.04.2018.A154 und 81-02.04.2019.A379). Eight heparinized pigs underwent graded carotid artery injury. Four pigs were subjected to 15 min of oversized balloon angioplasty only (Passeo-35, Biotronik, Berlin, Germany) with a balloon to artery ratio of 1.3:1. According to recent evidence, this injury leads to only subclinical vascular changes with an area stenosis of 10–20% after 4 weeks (BA, n = 4) [14]. The other four pigs underwent a combination of oversized balloon angioplasty and endothelial denudation using a Fogarty catheter. According to recent evidence, this injury leads a significant further decrease in lumen area and increased plaque size (BA + ECDN, n = 4) [15]. To this end, a 10 mm Fogarty over-the-wire embolectomy catheter (LeMaitre Vascular Inc., Burlington, MA, USA) was placed in one of the carotid arteries about 5 cm above the carotid artery trunk. The Fogarty balloon was then fully inflated and endothelial denudation was performed by pulling the inflated balloon down to the carotid artery trunk and repeating this procedure for a total of five times. The individual contralateral side served as internal control for each individual pig. Models were modified according to previous protocols [14, 15] and described in detail in the data supplement.

### Production, quality control and application of PFOB-nanoemulsion

Perfluorooctyl bromide-nanoemulsion (PFOB-NE) was produced according to established protocols [16]. In detail, 18.4 g purified egg lecithin (E 80 S, 4% *wt*/*wt*, Lipoid GmbH, Ludwigshafen, Germany) was dispersed in 285 g phosphate buffer (10 mM, pH 7.4) with 2.5% (v/v) glycerol by magnetic stirring at room temperature. Then 322 g PFOB (AtoChem, Puteaux, France) was added. Emulsions were stabilized by adding a semifluorinated alkane, which is a mixed fluorocarbon/hydrocarbon diblock compound (C_6_F_13_C_10_H_21_, *F*6*H*10; AtoChem, Puteaux, France) equimolar to the E 80 S lipid. Afterwards, the dispersion was pretreated with a high-performance disperser (T18 basic ULTRA TURRAX, IKA Werke GmbH & CO. KG, Staufen, Germany) at 14,000 rpm for 2 min. This pre-emulsion was further emulsified by high-pressure/shear homogenization (1000 bar, 30 min) using a microfluidizer (Microfluidizer M‐110P, Microfluidics Corp., Newton, MA, USA). Particle size was determined using photon correlation spectroscopy on a Zetatrac (Betatek, Toronto, Canada) device. Afterwards the PFOB-NE was autoclaved (30 min at 121 °C) using a program to autoclave pure liquids and stored at 4 °C for maximal 12 weeks until use. In addition, the emulsion was checked for the presence of particles ≥ 5 µm by light microscopy and each charge was tested for bacterial contamination.

PFOB-NE was administered intravenously at body weight adjusted dose of 5 ml/kg at day 3 after vascular injury according to the maximum circulating monocyte count and protocols adopted from experiments in myocardial infarction [12]. To this end, pigs were anaesthetized again as described above. PFOB-NE was applicated via a cannula placed in a superficial ear vein with an infusion rate of 100 ml/h. The mean infusion time was 4 h ± 31 min.

### Observation and assessment for severe adverse events or reactions

During infusion and in the course (24 h) following infusion, animals were monitored for potential side effects. Adverse reactions were defined as tachycardia, tachypnea, vomitus, rushing or abnormal behavior. Severe adverse reactions were defined as respiratory insufficiency with the need for re-intubation or death due to any cause.

### Invasive assessment of vascular morphology

**Fig. 1:**
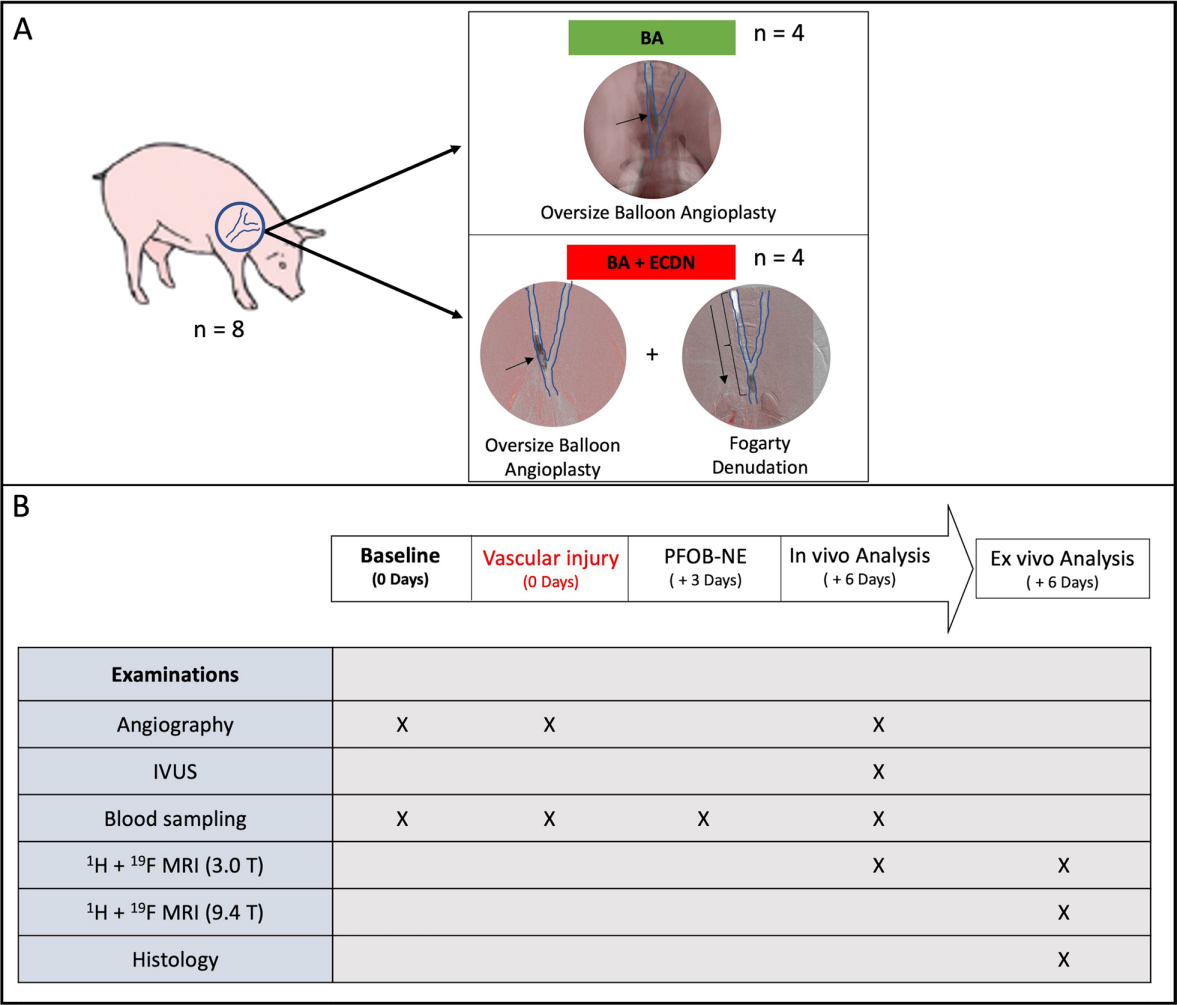
Experimental workflow and protocol. **A** Eight minipigs underwent either 15-min oversized balloon angioplasty (BA, n = 4) or oversized balloon angioplasty with additional endothelial denudation (BA + ECDN, n = 4) of the right carotid artery. **B** Vascular response to treatment was assessed by angiography at day zero. Perfluorooctyl bromide-nanoemulsion (PFOB-NE) was administered 3 days after injury. Vessel patency, analysis of the vascular wall and vessel blood flow was assessed by angiography and intravascular ultrasound (IVUS) at day 6. Afterwards, pigs were examined for vascular inflammation using in vivo and ex vivo ^1^H/^19^F MRI at 3.0 T and ex vivo high resolution ^1^H/^19^F MRI at 9.4 T followed by histology. BA = Balloon Angioplasty; ECDN = Endothelial Denudation; PFOB-NE, perfluorooctyl bromide-nanoemulsion

Before and seven days after vascular injury pigs were subjected to invasive assessment of carotid arteries. A detailed experimental flow chart and protocol is given in Fig. 1 and the data supplement. Briefly, carotid artery blood flow and vessel diameter were evaluated at baseline and 6 days after vascular injury by invasive angiography and intravascular ultrasound (IVUS). Serial blood samplings at day 1, 3 and 6 were obtained to investigate circulating inflammatory response to injury.

### ^1^H and ^19^F MRI data acquisition

Immediately after vascular assessment on day 6, non-invasive ^1^H- and ^19^F MRI was performed using a whole-body 3.0 T Achieva X-series scanner (Philips Healthcare, Best, the Netherlands). Anesthesia was maintained with a mixture of isoflurane (< 2.0% (v/v), Piramal Critical Care Deutschland GmbH, Hallbergmoos, Germany) in 100% oxygen. The MRI scan consisted of a ^1^H protocol including 3D time of flight (TOF) angiography, phase-contrast velocity encoded (VENC) measurements and high-resolution T2-weighted black blood sequences for vessel wall assessment. TOF angiography was performed using the following parameters: repetition time (TR) = 21.84 ms, echo time (TE) = 4.33 ms, flip angle (FA) = 16°. Furthermore, segmented gradient-echo phase contrast MRI was performed at the proximal, middle, and distal parts of the carotid artery (TR = 8.87 ms; TE = 5.22 ms; FA = 10°). The velocity encoding range was set at 150 cm/s in a through-plane direction. For vessel wall assessment a high-resolution T2-weighted black blood turbo spin echo sequences (TR = 1558 ms; TE = 60 ms; FA = 90°) was used. This was followed by a ^19^F protocol for monocyte/macrophage imaging with tested and optimized sequences [11]. After acquisition of ^1^H reference scans, the pigs were removed from the bore, and the ^19^F coil (7 × 12 cm dual tunable ^1^H/^19^F ellipsoidal coil (Philips Healthcare, Best, the Netherlands) was placed on the neck directly above the injured artery. The ^19^F ellipsoidal coil was tuned and matched for both ^19^F and ^1^H. ^1^H and ^19^F transmission could be switched by a small extra loop. Pigs were repositioned for optimal dataset overlay as previously established for local signal precision [11]. ^19^F imaging was performed using a balanced steady state free precession (bSSFP) sequence centered at 58 ppm (3D acquisition, with a 3 × 3 × 3 mm^3^ isotropic voxel size; TR = 2.88 ms; TE = 1.00 ms; FA = 30°), as described in a previous applicability study [10, 11]. Due to the frequency selective excitation at 58 ppm and the low isoflurane concentration applied (< 2.0% v/v) no relevant ^19^F signals from isoflurane could be observed in the lung or adipose tissue. After in vivo ^1^H- and ^19^F MRI acquisitions, pigs were euthanized with potassium chloride and Pentobarbital sodium (Narcoren^®^, Boehringer Ingelheim Vetmedica, Ingelheim, Germany).

### Autopsy, ex vivo MRI and histology

Autopsy was performed and both carotid arteries and the carotid trunk were excised *in toto* and stored in 4% paraformaldehyde (PFA) for at least 7 days. To optimize image conditions, but using the same technical equipment and sequences, ex vivo scans were performed at 3.0 Tesla with optimized coil distance of only a few mm and an identical isotropic image resolution for ^1^H and ^19^F (1 × 11 × mm^3^) with an image acquisition time of 19 min. To precisely localize ^19^F signals in carotid arteries for a direct histological validation, high resolution images of total excised carotid arteries were acquired at 9.4 T using a Bruker AVANCEIII 400 MHz Wide Bore NMR spectrometer (Bruker, Rheinstetten, Germany). Here, ^19^F datasets were recorded using a 3D RARE sequence (RARE factor 32, TR 3.5 s, 330 × 450 µm in-plane resolution, Slice thickness 1 mm, 465 averages, Scan time 48 h). For exact anatomic localization, the ^19^F datasets were merged with corresponding 3D 1H RARE scans (RARE factor 16, TR 5 s, 120 × 120 µm in-plane resolution, Slice thickness 1 mm, 10 averages, Scan time 16 h).

Subsequently paraffin embedded vessels were sectioned (5 µm) and stained with hematoxylin/eosin (H/E). For immunoflorescence and immunohistology the following antibodies were used: anti-CD163 antibody (clone 2A10/11, Bio-Rad, Hercules, CA, USA) for identification of M2-like macrophages; anti-CD68 antibody (ab125212, 7.5 µg/ml, abcam, Cambridge, UK) for identification of M1-like macrophages. Tissue processing is described in detail in the data supplement. Histological examination was conducted with a Leica microscope (DM 4000 M, Leica Microsystems, Wetzlar, Germany) with 10 ×, 20 × and for detailed images 40 × objectives. H/E- or CD163/CD68-stained cells were counted semi-automatically using ImageJ (LOCI, WI, USA) with a protocol provided in the data supplement. Early Remodeling Index (RI) was calculated by using histology: RI was defined as lesion external elastic membrane cross-sectional area (EEM CSA) divided by mean reference EEM CSA.

### MRI data analysis

MRI datasets were visualized and analyzed for signal overlays using HOROS (Nimble Co LLC, Annapolis, MD, USA) and Circle CVI (Circle Cardiovascular Imaging Inc., Calgary, Canada) for analysis of anatomic dimensions and velocities. For assessment of in vivo ^19^F datasets, exact anatomical co-localization was performed by image fusion of ^1^H and ^19^F datasets using HOROS. For quantification of ^19^F signal intensity, the primary signal at a respective region was corrected according to the coil sensitivity profile: $SNR_{Z \,direction} = 0.45 \cdot SNR_{measured} /e^{ - Distance \,in \,Z \,direction/75 mm}$ [12]. Then, SNRs were calculated from the ratio of the mean of a region of interest (ROI) and the standard deviation of the noise of a ROI outside the body [11, 12]. ^19^F volumes were calculated by applying background subtraction with SNR = 5 which was sufficient to subtract all unspecific technical background signals (outside the body). For validation of ^19^F signals with histology at least 10 segments of each excised carotid artery were analyzed for ^19^F SNR and histology (n = 80 segments). The regions were identified by measuring the distance from bifurcation to the respective region in cm.

As minimal detection limit, we defined the minimal number of monocytes/macrophages per mm^2^ that correlates to a visible ^19^F Signal (as defined as signal-to-noise ration > 5) in every single sample. However, as there are samples with even lower monocyte/macrophage cell count leading to a ^19^F SNR > 5, the actual detection limit might even be lower.

### Statistical analysis

The detection limit of inflammatory cells using ^19^F MRI at 3.0 Tesla was previously determined 400 macrophages/mm^2^ in a model of myocardial infarction [5]. This detection limit was considered suitable for imaging vascular inflammation in models of mechanical injury and even human atherosclerotic plaques [17].

For statistical analysis of histology, each evaluated carotid artery was cut into at least 10 cross sections. Histological analysis was performed separately in all resulting sections. Afterwards, a mean value for each carotid artery was calculated. The final statistical analysis (vessel wall thickness, monocyte/macrophage cell count, remodeling index) was then carried out using one average value per carotid artery (n = 4 for BA and BA + ECDN, n = 8 for control carotid artery).

Statistical analysis was performed using GraphPad Prism (Version 9, IBM, San Diego, CA, USA). Unless otherwise stated, continuous variables are presented as mean ± standard deviation (SD). Normal distribution was tested using the Shapiro–Wilk test. Data between the two different groups (BA and BA + ECDN) were analyzed by 2-sided unpaired Student’s t-tests for normally distributed data and Mann–Whitney U-test for not normally distributed data. Data comparing three groups (Control vs. BA vs. ECDN) were analyzed using a one-way ANOVA followed by Šidák multiple comparison test. Pearson’s correlation was used to assess the relationship between different ^19^F signal intensity and monocyte/macrophage cell count.

## Results

### Experimental workflow and safety of PFOB-NE application

Eight minipigs underwent graded carotid artery injury by either 15 min of oversized balloon angioplasty known to induce only subclinical stenosis [14] (BA, n = 4) or a combination of 15 min oversized balloon angioplasty and mechanical denudation (BA + ECDN, n = 4) using a Fogarty catheter known to cause clinical significant stenosis (Fig. 1) [15]. All animals survived the procedure. Blood sampling and angiography was performed before and after vascular injury. At day three after injury, PFOB-NE was administered intravenously. In 4 of 8 pigs, adverse reactions were observed, but no severe adverse reaction occurred (Additional file 1: Table S1). At day 6 after injury blood sampling as well as invasive angiography and IVUS was performed followed by in vivo and ex vivo ^1^H- and ^19^F-MRI and histology. Numbers of circulating leukocyte increased mildly from baseline to day 6 after vascular injury and hemoglobin levels remained constant (Additional file 1: Table S2).

### Effective induction of graded vascular injury and early remodeling 

**Fig. 2:**
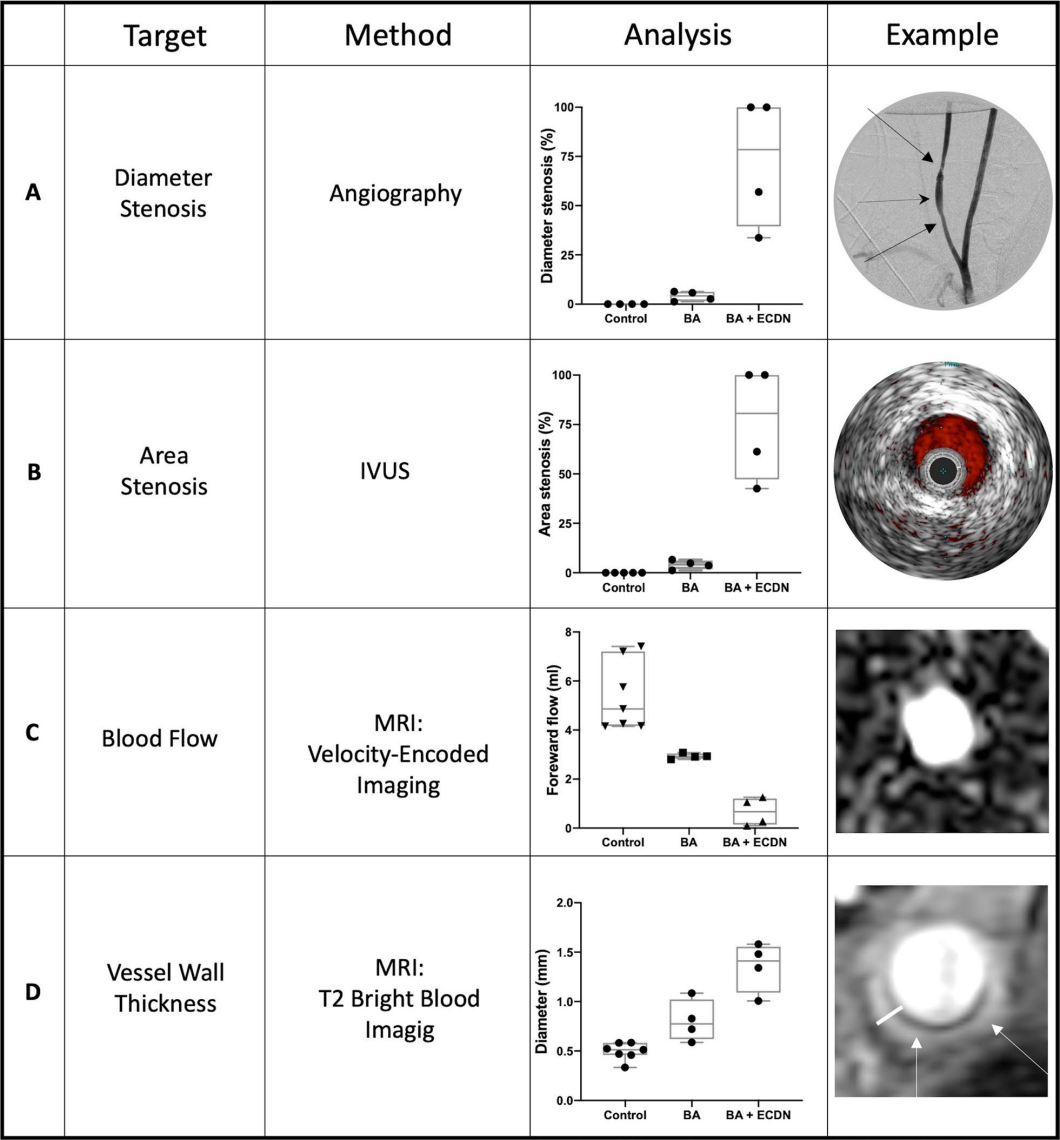
Angioplasty-induced morphological and functional vascular adaptations. Carotid artery stenosis was evaluated using invasive angiography (**A**) and IVUS (**B**). Velocity-encoded MRI was used to quantify carotid blood flow (**C**) and vessel wall thickness was measured in MR-Angiography (**D**). Examples of the resulting images after BA + ECDN are shown on the right. BA + ECDN induced a relevant carotid artery stenosis or occlusion accompanied by reduction in blood flow and increased vessel wall thickness. BA = Balloon Angioplasty; ECDN = Endothelial Denudation

At day 6 after injury, angiographic assessment and IVUS revealed no relevant stenosis after BA. However, there was a 56% and 33% diameter stenosis with maintained blood flow in two pigs after BA + ECDN while two pigs displayed nearly total carotid artery occlusion (Fig. 2A). Carotid artery blood flow was assessed non-invasively by VENC MRI (Fig. 2B). Following BA + ECDN, a decrease in forward flow and post stenotic maximum velocity could be detected in all four pigs. Two pigs showed signs of nearly total artery occlusion with a decrease in forward flow and maximal velocity to nearly zero. Nevertheless, histological analysis of the vessel wall including thickness, remodeling index and tissue composition with inflammation was possible. While vessel wall thickness was approximately 0.8 mm after BA, BA + ECDN led to an increase to 1.4 mm at day 6 as quantified non-invasively using high-resolution T2-weighted bright blood imaging (Fig. 2C).

### Early vascular remodeling after vessel injury

**Fig. 3:**
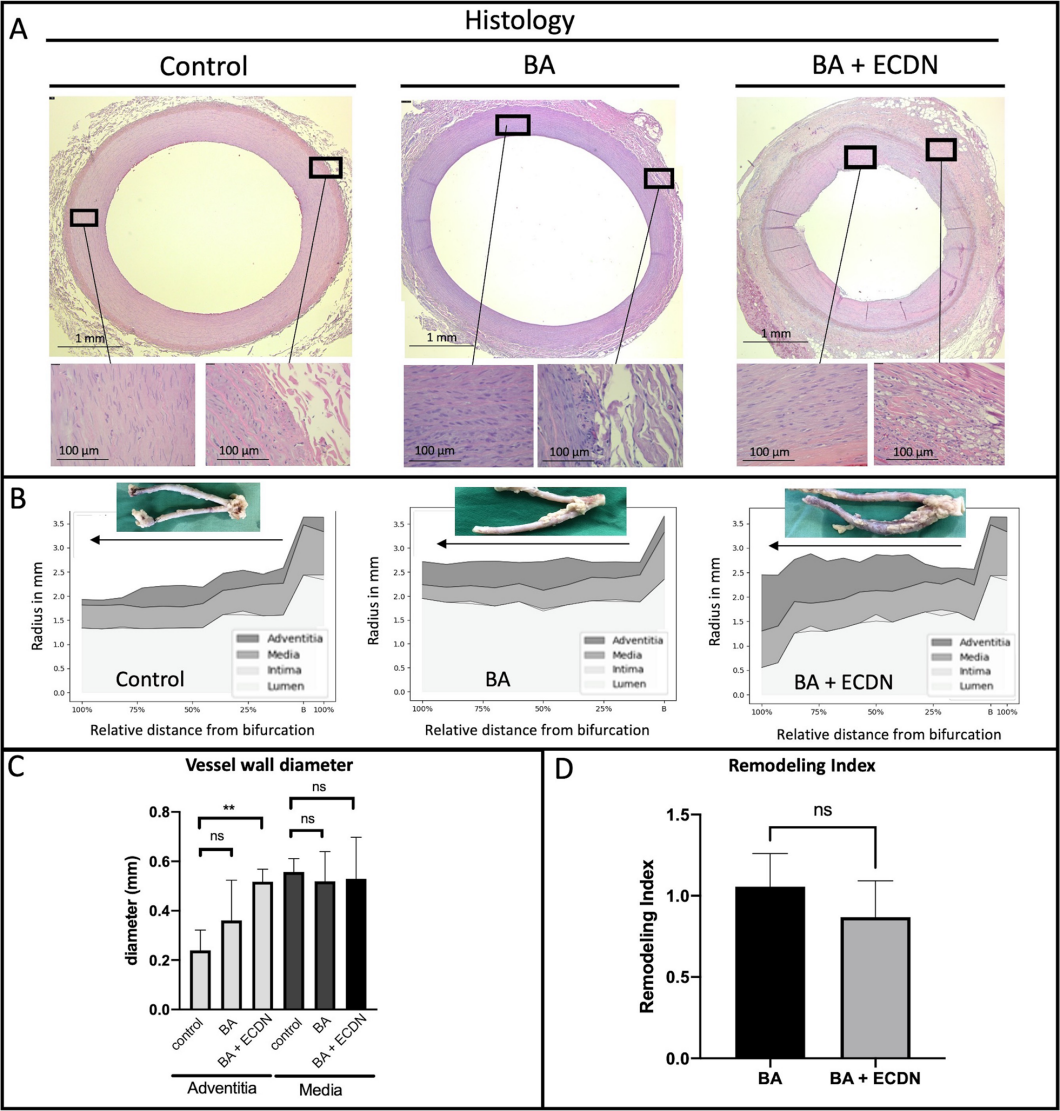
Histological analysis of carotid artery after angioplasty. Explanted carotid arteries of all 8 animals were cut into at least 10 sections. Analysis was performed in every resulting histologic section. **A** Typical examples of HE-stained histological sections six days after graded vascular injury. **B** Vascular lumen, intima, media and adventitia thickness along the entire vessel length as given in relative distance from bifurcation. Macroscopic images of the analyzed vessel are shown above. **C** Measurements of vascular wall thickness of media and adventitia in BA and BA + ECDN as well as contralateral control carotid artery. **D** Remodeling index (External elastic lamina area diseased/control) reveals no significant difference after BA and BA + ECDN. BA = Balloon Angioplasty; ECDN = Endothelial Denudation; ns = not significant, *p < 0.05, ***p < 0.001

H/E staining was performed to quantify early vascular remodeling after graded vessel injury. Mean diameter of lumen, intima, media, and adventitia was measured in at least 10 cross-sections starting from the carotid artery bifurcation and expressed as relative distance from bifurcation (Fig. 3A and B). Adventitial diameter increased compared to contralateral control carotid arteries after BA and BA + ECDN while diameter of media and intima remained unaffected. Only after BA + ECDN a relevant reduction of luminal radius was observed. Quantification of vessel wall diameter across a range of histological sections obtained from all 8 pigs verified these findings in the individual arteries (Fig. 3C). Compared to contralateral control carotid arteries, adventitial wall thickness increased more than twofold after BA + ECDN, while media thickness was not significantly changed. Remodeling index (external elastic lamina area diseased/control) showed no significant difference after BA + ECDN compared to BA (Fig. 3D).

### ^19^F MRI reveals focal spots of macrophages after injury

**Fig. 4:**
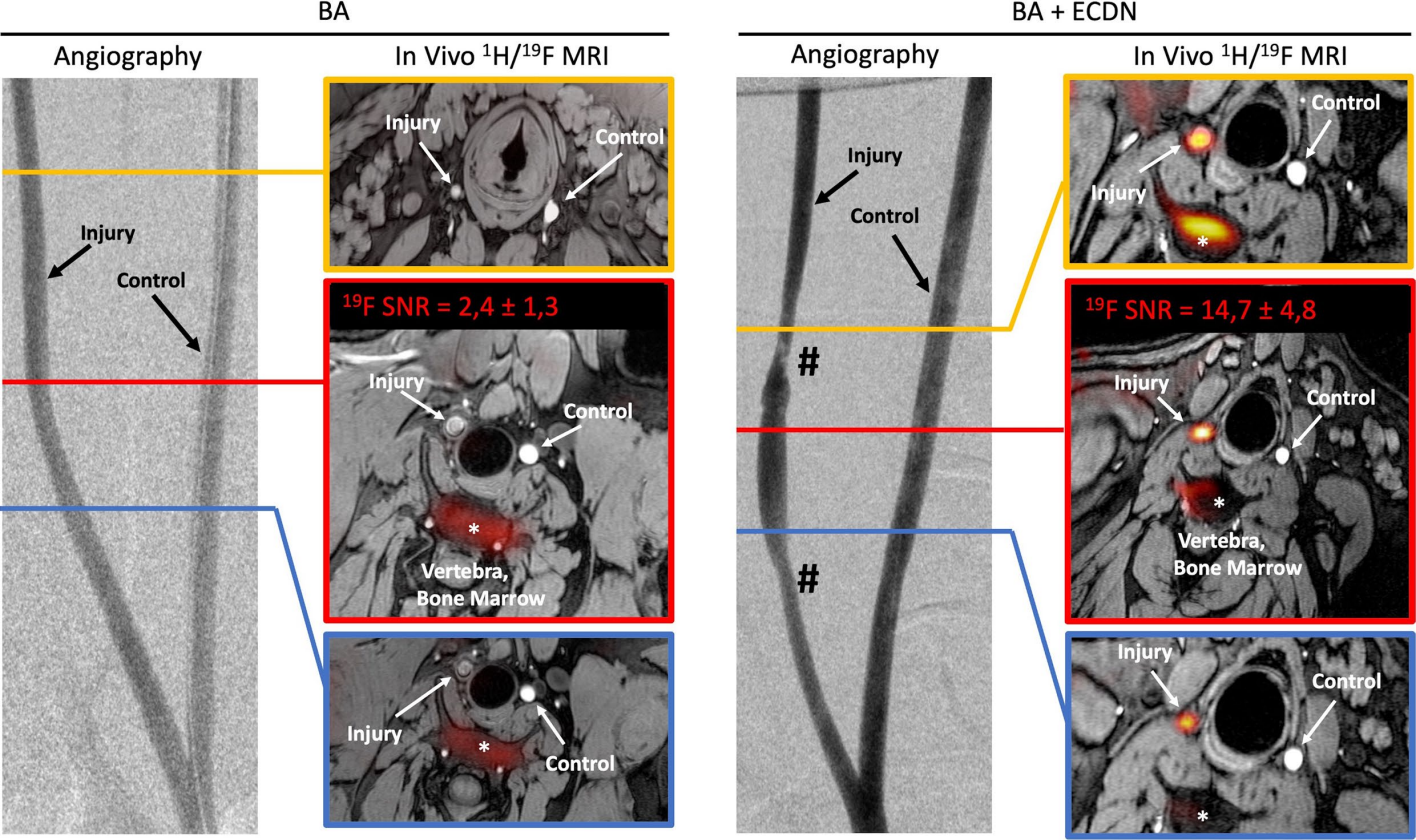
BA + ECDN induces a robust ^19^F signal in vivo. Pigs (n = 8) were assessed for vascular ^19^F signal 6 days after graded vessel injury. Angiographic images with indicated angioplasty regions of interest. Note vascular stenosis (#) in the images of BA + ECDN. For anatomical in vivo co-localization ^19^F images were fused with ^1^H 3D-T1 weighted images. ^19^F signal intensity is shown on a ‘hot iron’ color scale. After BA + ECDN, a robust ^19^F signal was detected in all pigs (right panels, n = 4) in vivo. No in vivo ^19^F signal was found in pigs following BA alone (left panels, n = 4). BA = Balloon Angioplasty; ECDN = Endothelial Denudation; SNR = Signal-to-noise ratio

To assess inflammatory processes in addition to anatomical ^1^H MR images, ^19^F images were displayed on a ‘hot iron’ color scale and fused with ^1^H series to ensure exact anatomical localization of ^19^F signal. These analyses were performed in vivo and validated by high-field MRI ex vivo (Fig. 4).

Following BA + ECDN, a robust in vivo ^19^F signal with a SNR of 14.7 ± 4.8 after 19 min of image acquisition was detected in the treated carotid artery of all 4 pigs (Fig. 4). No signal was found in the contralateral control carotid artery. Vascular ^19^F signal was clearly distinct from other tissue signals, mainly derived from the bone marrow and subcutaneous fat. No vascular ^19^F signal was found in vivo after BA-treatment alone. In the ex vivo setting at 3 T, ^19^F signal could be detected after 20 min in all 8 pigs with a strong SNR of 23.4 ± 3.8 after BA + ECDN and weak SNR of 6.2 ± 1.2 following BA. In vivo and ex vivo imaging revealed a patchy distribution of ^19^F signal along the carotid artery.

### Local ^19^F signal co-registers quantitatively with accumulation of monocytes/macrophages

**Fig. 5:**
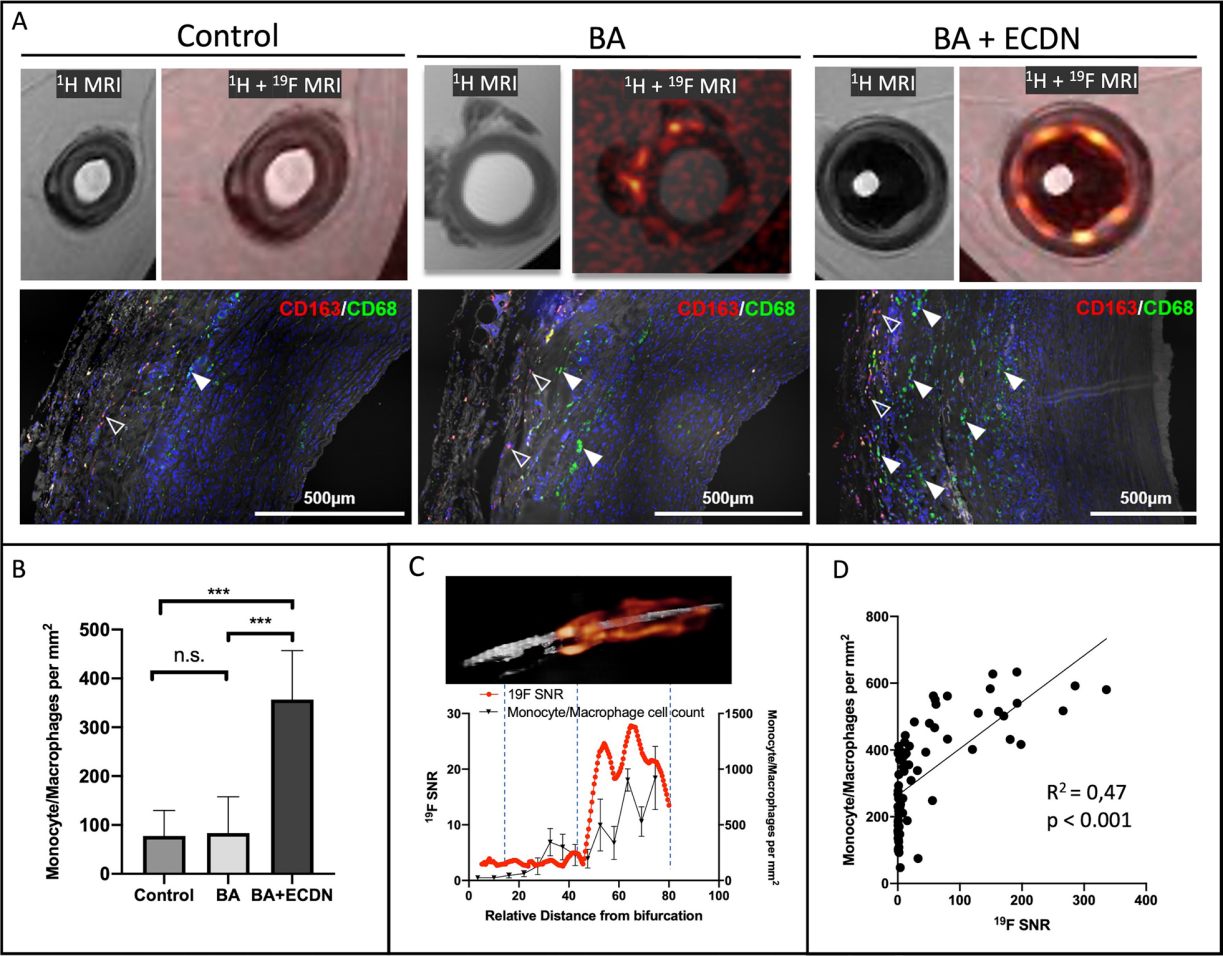
Local ^19^F signal co-localizes with accumulation of monocytes/macrophages. High-resolution ex vivo ^1^H and fused ^1^H/^19^F MR images at 9.4 T show a robust ^19^F signal derived from the vascular wall after injury. Immunofluorescence staining using CD163 (red, open arrowheads) and CD68 (green, filled arrowheads) antibody confirmed a predominant adventitial infiltration of monocytes/macrophages. **A** Monocytes/macrophages were counted from at least 10 histological cross sections per artery showing a large increase after BA + ECDN while the increase after BA remained only minor (**B**). Local ^19^F SNR measurements at 3 T were performed cross-sectional in correspondence to histologic cell count revealing an exact co-localization along the whole length of the artery (**C**) and ^19^F signal intensity correlates to monocyte/macrophage count (**D**). BA = Balloon Angioplasty; ECDN = Endothelial Denudation; SNR = Signal-to-noise ratio

To validate the patchy appearance of ^19^F signals and to define minimal ^19^F detection limits for monocytes/macrophages in vascular inflammation, we conducted ex vivo optimized ^1^H- and ^19^F-imaging of excised carotid arteries at 3 T and high-resolution ex vivo ^1^H- and ^19^F-imaging at 9.4 Tesla alongside histological monocytes/macrophages staining (Fig. 5A). Fusion of high resolution ^1^H /^19^F images clearly demonstrated that PFOB-derived ^19^F signal stems from the vascular wall and that the ^19^F signal appears in a patchy fashion. Immunofluorescence staining confirmed a corresponding adventitial presence of monocytes/macrophages with predominantly CD163^−^CD68^+^ and to a lesser extent CD163^+^CD68^−^ appearance.

Semi-automated counting of monocytes/macrophages revealed a 4.6-fold increase of monocytes/macrophages in the adventitia after BA + ECDN while only a minor increase could be detected after BA (Fig. 5B). In the media, monocytes/macrophages were sparsely detected after both BA and BA + ECDN, suggesting that monocyte/macrophage infiltration early after vascular injury mainly occurs via the adventitia. The adventitial monocytes/macrophages phenotype was a mixture of CD68^+^ CD163^−^ and CD68^−^ CD163^+^ cells with predominance of CD68^+^ CD163^−^ in BA + ECDN.

For histological validation carotid arteries were cut into at least 10 segments. Co-registration of local ex vivo ^19^F signal at 3.0 T with segmental monocytes/macrophages cell count was performed over the entire length of the carotid arteries of every included pig (n = 8) leading to about 80 data points. This analysis of individual arteries revealing a close relationship between local ^19^F signal intensity as detected by MRI and spots of monocyte/macrophage accumulation (Fig. 5C). A significant correlation (R^2^ 0.47, p < 0.001) of local ^19^F signal with monocyte/macrophage cell density was found when analyzing every segment of the injured carotid arteries after BA as well as BA + ECDN. The detection limit was about 410 monocytes/macrophages per mm^2^.

## Discussion

This present study shows that (1) imaging carotid artery inflammation after mechanical injury with ^19^F MRI is feasible within 19 min using a clinical MR scanner and a human-sized model; (2) local ^19^F signal quantitively co-registers with accumulation of monocytes/macrophages and (3) ^19^F signal reflects predominately early adventitial infiltration of CD163^−^ CD68^+^ monocytes/macrophages associated with vascular wall thickening.

### Feasibility of PFOB-derived ^19^F MRI to detect vascular monocytes/macrophages

Experimental approaches in mice have proven feasibility of ^19^F MRI to quantitatively visualize macrophage accumulation in a wide range of experimental set-ups and disease models, including vascular inflammation, [5, 6, 18]. However, those experiments were conducted with Perfluorpolyether (PFPE), which confers excellent imaging properties. Yet, a long body half-life renders it less attractive for a potential clinical application. In contrast, other PFCs (e.g., PFOB) have a shorter biological half-life and have already proven safe and efficient applicability in clinical trials for testing their oxygen transport capacities [19]. Thus, these compounds might have greater potential for clinical translation. Furthermore, ^19^F MRI using PFOB nanoemulsion is also feasible in pre-clinical models after myocardial infarction using clinical scanners [10–12] with good agreement between hot spots of ^19^F signal and macrophage accumulation [12]. In the present study, we used PFOB for ^19^F MRI in an animal model with a body size and an inflammatory reaction comparable to humans to study macrophage visualisation after vascular injury. The resulting ^19^F signal in carotid arteries after balloon injury was highly reproducible with a robust in vivo and ex vivo SNR three or five times above the common detection threshold (SNR = 5). Accordingly, our study provides evidence that ^19^F MRI using PFOB-nanoemulsion could be a valuable tool to monitor vascular monocytes/macrophages accumulation after angioplasty.

### Safety of PFOB derived ^19^F MRI

The application of PFOB-NE appeared overall safe corroborating a previous study in a model of myocardial infarction that did not reveal severe adverse reaction (Additional file 1: Table S1). In line with this, the infusion of a significant larger amount of the emulsion did not lead to increased mortality in a clinical phase 3 trial [19]. In the present study we conducted a rigorous quality control of each emulsion charge with careful assessment of particle size. Moreover, the analysis of circulating leukocyte indicated no severe systemic inflammatory reaction upon PFOB infusion and no drop in hemoglobin levels (Additional file 1: Table S2).

### Cellular source of PFOB-derived ^19^F signal

There is accumulating evidence, that tissue macrophages are the main source of PFOB-derived ^19^F signals, as shown in mice [5], pigs [10–12] and humans [7]. Untargeted perfluorocarbon nanoemulsions are taken up by classical and alternatively-activated macrophages to an equal extent [20]. Minimal detection limits were calculated to be around 400 macrophages/mm^2^ tissue utilizing a model of myocardial infarction [5]. In the present study, we used a model of oversized balloon angioplasty with or without additional Fogarty-induced mechanical denudation (BA vs. BA + ECDN) according to established protocols [15]. A reliable and reproducible in vivo ^19^F signal could only be detected after BA + ECDN. In our study, the minimal detection threshold for detection of macrophages was about 410 cells/mm^2^ corroborating previous estimation [5]. The signal was clearly restricted to the vascular wall. Although 2 animals developed a luminal thrombosis, all 4 animals of this group could be analyzed for the PFOB-derived ^19^F signal that was confirmed histologically to stem from macrophage infiltrates.

Untargeted perfluorocarbon nanoemulsions are taken up by classically- (M1-like) and alternatively-activated (M2-like) tissue macrophages to an equal extent [20]. Given the fact, that M1-like macrophages were present in larger numbers in our model on day 6 after angioplasty, the observed ^19^F-signal reflected most likely M1-like macrophages. This finding mirrors recent evidence in pigs after myocardial infarction [12].

In addition to profound macrophage infiltration, BA + ECDN-induced injury was consistently paralleled by adventitial wall thickening. In contrast, BA induced only a sparse infiltration of monocytes/macrophages in the vascular wall detected by immunohistology. This was paralleled by a mild increase in adventitial wall thickening. The signal of those inflammatory cells was only measurable under optimal coil distance conditions using ex vivo ^19^F scans. In both groups (BA vs. BA + ECDN), there was no significant difference in the vascular remodeling index, underlining the predominant adventitial remodeling, as this index mainly reflects changes in the intima and media.

Other studies using balloon oversize injury in pigs mainly focused on intimal hyperplasia, neointima formation and lumen area after a follow up period of 2–4 weeks [14, 15]. Nevertheless, these studies confirm subclinical early vascular remodeling after overstretch injury with minimal reduction in lumen area [14]. In contrast, balloon oversize injury followed by endothelial denudation caused significantly reduced lumen area and enhanced early vascular remodeling [15]. Therefore, mild monocyte/macrophage infiltration below the ^19^F detection limit may also be of minor significance for early vascular remodeling and subsequent development of re-stenosis.

### Using ^19^F MRI to identify individuals at risk

There is evidence, that identification of vessel wall surrogates of inflammation with PET (glucose uptake (^18^F-FDG) or microcalcification (^18^F-NaF)) can predict restenosis after angioplasty [21]. We have shown, that ^19^F MRI with an untargeted nanoemulsion can directly image and quantify macrophage infiltration in vascular inflammation [4, 22]. The specificity of this approach to target classically- and alternatively-activated macrophages could even be improved by functionalizing the nanoemulsion [4, 22]. As previously demonstrated in mice, multitargeted nanoemulsions with different ^19^F-agents identified by multi-chemical shift-selective imaging are a powerful tool to monitor simultaneously a broad range of antigens important in the development and exacerbation of atherosclerosis (“multi-color-imaging”) [4]. Thus, the present imaging platform may provide the basic methodology for a comprehensive phenotyping of vascular healing after angioplasty with the major advantage of a positive, specific and quantifiable signal. In the present study, using an untargeted nanoemulsion, we could directly and quantitatively visualize the monocyte/macrophage lesion burden with a resolution sufficient for improved vascular-wall mapping of the signal.

Interestingly, the minimal detection limit of ^19^F MRI was comparable to cell density of human carotid atherosclerotic macrophage-rich vulnerable plaques [3, 23]. As shown for primary prevention, the indirect visualization of inflammatory foci by enhanced glucose uptake using ^18^F-fluorodeoxyglucose (^18^F-FDG) predicts subsequent vascular events in carotid artery atherosclerotic lesions [24, 25]. Inflammatory microcalcification as identified by ^18^F-sodium-fluoride (^18^F-NaF) can predict future cardiovascular events in the coronary arteries [26, 27]. Given the fact, that the number of monocytes/macrophages is directly related to plaque vulnerability [3, 28], quantitative imaging of monocytes/macrophages may provide additional value for identifying the patient at risk for imminent clinical complication.

### Future direction

Promising advances in MRI-technology such as improved coil designs and MRI sequence optimization have the potential to enhance the sensitivity of this methodology. The current timing of PFOB-NE administration and ^19^F MRI imaging was adopted from our protocol of acute myocardial infarction [11, 12]. Since the time course of cellular response to vascular injury may differ from that in myocardial infarction, optimizing time point and duration of PFOB-NE administration and ^19^F MRI imaging might even increase sensitivity of the method. In addition, the PFOB-load of the individual nano-droplet could be increased. Furthermore, human monocytes are even more capable of PFOB phagocytosis than those from mice or pigs [7, 10, 12] and remain functional thereafter. Finally, targeted nanoemulsions against specific epitopes can be PEGylated, thus rendering them more potent to pass the bone marrow [4]. Accordingly, they may be longer available to the target cells, thus leading to an additional signal increase. Future studies should investigate the dynamic signal flux that is expected by the dynamic monocyte/macrophage turnover.

### Study limitations

The present study only evaluated acute vascular inflammation. Due to the validating nature of the present study, there were no sequential scans which would have possibly given insight in the signal dynamics over time. We cannot make a conclusive statement regarding the significance of ^19^F signal intensity for long-term vascular remodeling or chronic inflammatory processes in atherosclerosis-driven peripheral artery disease. Further long-term studies with graded vascular injury and large animal dietary or genetic models of atherosclerosis are needed to investigate vascular remodeling and plaque progression in relation to ^19^F signal intensity.

## Conclusion

^19^F MRI enables quantification of monocyte/macrophage infiltration after vascular injury with sufficient sensitivity. This might open an avenue to non-invasively monitor vascular inflammation with MRI to either predict angioplasty-derived restenosis or atherosclerotic plaque inflammation.

### Supplementary Information

**Additional file 1. **Supplemental Material.

## Data Availability

Original data are available upon reasonable request.

## Abbreviations

^19^F MRI: Fluorine-19 magnetic resonance imaging
BA: Balloon oversize angioplasty
BA + ECDN: Balloon oversize angioplasty with endothelial denudation
DEB: Drug-eluting balloon
DES: Drug-eluting stent
EEM: External elastic membrane
H/E: Hematoxylin/Eosin
IVUS: Intravascular ultrasound
PAD: Peripheral artery disease
PFA: Paraformaldehyde
PFC-NE: Perfluorocarbon-nanoemulsions
PFOB-NE: Perfluorooctyl bromide-nanoemulsion
PFPE: Perfluorpolyether
RI: Remodeling index
ROI: Region of interest
SNR: Signal-to-noise ratio
TOF: Time of flight angiography
