# Supplementary material for: Quantitative assessment of angioplasty-induced vascular inflammation with 19F cardiovascular magnetic resonance imaging
Source: J Cardiovasc Magn Reson. 2023 Oct 3;25:54. doi: 10.1186/s12968-023-00964-7 (PMC10546783; doi:10.1186/s12968-023-00964-7)
Supplement: Supplementary file 1 — Additional file 1. Supplemental Material. [file 12968_2023_964_MOESM1_ESM.docx]

**Supplement**

**Quantitative assessment of angioplasty-induced vascular inflammation with**

**^19^F cardiovascular magnetic resonance imaging**

Short title: Quantitative Imaging of Vascular Inflammation

Fabian Nienhaus^a^, Moritz Walz^a^, Maik Rothe^b,c^, Annika Jahn^a,d^, Susanne Pfeiler^a^, Lucas Busch^a^, Manuel Stern^a^, Christian Heiss ^I,k^, Lilian Vornholz^a^, Sandra Cames^b,c^, Mareike Cramer^a^, Vera Schrauwen-Hinderling^b,c^, Norbert Gerdes^a,g^, Sebastian Temme^e,h^, Michael Roden^b,c,f^, Ulrich Flögel^e,g^, Malte Kelm^a,g^, Florian Bönner^a,g^

^a^Division of Cardiology, Pulmonology and Vascular Medicine, University Hospital and Medical Faculty, Heinrich-Heine-University, Düsseldorf, Germany

^b^ Institute for Clinical Diabetology, German Diabetes Center, Leibniz Center for Diabetes Research at Heinrich Heine University, Düsseldorf, Germany

^c^ German Center for Diabetes Research, Partner Düsseldorf, Germany

^d^ Central animal research facility, Heinrich Heine University, Düsseldorf, Germany

^e^ Experimental Cardiovascular Imaging, Department of Molecular Cardiology, Medical Faculty, Heinrich-Heine-University Düsseldorf

^f^Division of Endocrinology and Diabetology, Medical Faculty, Heinrich Heine University, Düsseldorf, Germany

^g^ Cardiovascular Research Institute Düsseldorf (CARID), Medical Faculty, Heinrich-Heine-University, Düsseldorf, Germany.

^h^Experimental Anesthesiology, Medical Faculty, Heinrich Heine University, Düsseldorf, Germany

^i^ Department of Clinical and Experimental Medicine, University of Surrey, Faculty of Health and Medical Sciences, Guildford, United Kingdom

^k^ Department of Vascular Medicine, Surrey and Sussex Healthcare NHS Trust, Redhill, United Kingdom

**Supplemental Material and Methods**

**Animals**

The experiments were performed in 8 adult Aachen minipigs with a medium age of 2 years old (2 years ± 5 months). Pigs were obtained from a local breeder and housed at the central animal facility center of Heinrich-Heine-University, Düsseldorf, Germany. All study protocols were carried out in accordance to the national guidelines on animal care and approved by the state authority `Landesamt für Natur-, Umwelt- und Verbraucherschutz‘.

**Anesthesia and analgesia**

Pigs were fasted overnight before surgery. While in their quarters, pigs were sedated with Stresnil® (5mg/kg BW IM, Elanco Lilly Deutschland GmbH, Bad Homburg, Germany) followed by Ketamin (10 mg/kg BW IM, Ketaset Zoetis, Berlin, Germany) and Atropinsulfat (0,5mg IM, Braun, Melsungen, Germany) with an additional dosage of Diazepam (10mg IM, Ratiopharm GmbH, Ulm, Germany). After 10-20 minutes a cannula was introduced into a superficial vein of the ear and complete anesthesia was induced with sodium thiopental (4mg/kg IV initially and then demanding on effect, Rotexmedica GmbH, Trittau, Germany). Intubation of the pigs was performed orotracheal using a size-matched tube (7,5-8,5 mm tube) with pigs being in supine position. Anesthesia was maintained with a mixture of isoflurane (1,5-2,0 Vol %, Piramal Critical Care Deutschland GmbH, Hallbergmoos, Germany) solved in 100% oxygen. Pigs were ventilated with a respirator (Sulla 808 V, Dräger, Lübeck, Germany) at a rate of 10 to 12 breaths per minute, tidal volume was 450 ml (range 400-550ml). For cooling protection, the animals were positioned in supine position on a heating mat on the operating table. Biomonitoring was assured by continuous monitoring of ECG, blood pressure, oxygen saturation (Monitor Eagle 4000, Marquette Heilige GmbH, Freiburg, Germany) and intermittent analysis of arterial blood gases. To maintain, preload stability, normal saline (2mL/kg hourly, Braun, Melsungen, Germany) was infused through the venous cannula in the auricular vein during surgery. Every 20 to 30 minutes the pigs received a bolus of Fentanyl thereby maintaining analgesia (0,0075mg, Rotexmedica GmbH, Trittau, Germany).

**Model of Vascular Injury**

Vascular injury was induced in all animals in one carotid artery with a combination of Fogarty balloon denudation and balloon oversize injury modified according to previous protocols [1-4]. In detail, the femoral artery was exposed by surgical incision and preparation under sterile conditions. A 6F introducer sheat was inserted into the artery followed by administration of a heparin bolus (100 IU/kg). A guidewire (Emerald Guidewires 260 cm 0.032 inch, Cordis, Santa Clara, CA, USA) was placed in the carotid artery trunk and pre-injury carotid angiography was obtained by rapid administration of a iodine-containing contrast agent (Accupaque©, General Electronic Healthcare, Solingen, Germany) using a 6F JR 4.0 SH guiding catheter (Medtronic, Inc., Minneapolis, MN, USA). Four pigs were subjected to 15 min of oversized balloon angioplasty only (Passeo-35, BIOTRONIK, Berlin, Germany) with a balloon to artery ratio of 1.3 : 1 known to induce only subclinical vascular changes with an area stenosis of 10-20% after 4 weeks (BA, n=4). In additional n=4 animals a 10 mm Fogarty over-the-wire embolectomy catheter (LeMaitre Vascular Inc., Burlington, MA, USA) was placed in one of the carotid arteries about 5 cm above the carotid artery trunk. The Fogarty balloon was then fully inflated and endothelial denudation was performed five times in a row by pulling the inflated balloon down to the carotid artery trunk. Afterwards a Passeo 35 over-the-wire angioplasty balloon (10/40 mm, Biotronik, Berlin, Germany) was placed in the denudated area and oversize injury was performed three times in a row by fully inflation of the balloon to 7 atm for each 5 min. Post-injury angiography was obtained again as described above. After surgery all external material was removed and the wound was closed.

**Production of PFOB-Nanoemulsion**

PFOB-NE was prepared as described elsewhere [5-7]. Briefly, 18.369 g purified egg lecithin (E 80 S, 4% *wt*/*wt*, Lipoid GmbH, Ludwigshafen, Germany) was dispersed in 284.508 g phosphate buffer (10 mM, pH 7.4) with 2.5% Glycerol by magnetic stirring at room temperature. Then 322 g PFOB (AtoChem, Puteaux, France) was added. Emulsions were stabilized by adding a semifluorinated alkane, which is a mixed fluorocarbon/hydrocarbon diblock compound (C_6_F_13_C_10_H_21_, *F*6*H*10) equimolar to the E 80 S lipid. Afterwards, the dispersion was pretreated with a high-performance disperser (T18 basic ULTRA TURRAX, IKA Werke GmbH & CO. KG, Staufen, Germany) at 14000 rpm for 2 min. This pre-emulsion was further emulsified by high-pressure/shear homogenization (1000 bar, 30 min) using a microfluidizer (Microfluidizer M‐110P, Microfluidics Corp., Newton, MA, USA). Particle size was determined using photon correlation spectroscopy (PCS) on a Zetatrac (Betatek, Toronto, Canada) device. Afterwards the NE was autoclaved (30 min at 121°C) using a program to autoclave pure liquids and stored at 4°C.

**Application of PFOB-Nanoemulsion**

At day 3 after vascular injury a body weight adjusted volume of PFOB-NE (5 ml/kg body weight) was administered intravenously. Therefore, pigs were anaesthetized again as described above. PFOB-NE was applicated via a cannula placed in a superficial ear vein with an infusion *rate of 100 ml/h.*

## **Observation and Assessment for severe adverse events or reactions**

During infusion and over 24 hours following infusion, animals were checked for adverse and severe adverse reactions. Adverse reactions were defined as tachycardia, tachypnea, vomitus, rushing or abnormal behavior. Severe adverse reactions were defined as respiratory insufficiency with the need for re-intubation or death.

**Invasive assessment of vascular injury**

At day 6 after surgery, carotid artery injury was assessed invasively by angiography and intravascular ultrasound (IVUS). Therefore, pigs were anaesthetized again and angiography of the carotid arteries were obtained as described above. The degree of carotid artery stenosis was determined by calculation of diameter stenosis. Blood flow was assessed visually. Afterwards, an IVUS catheter was introduced into the carotid artery via a 0.035 inch balanced middleweight (BMW) guidewire (Abbott Laboratories, Chicago, Illinois, USA). IVUS and colour flow IVUS was performed in both the injured and the non-injured artery. Lumen size and Neointima thickness was measured. Blood flow and wall abnormalities were analyzed visually.

**^1^H and ^19^F Cardiovascular Magnetic Resonance (CMR)**

For cardiovascular magnetic resonance (CMR), pigs were anesthetized as described above. Anesthesia was maintained with a mixture of isoflurane (1.5 – 2.0 % v/v, Piramal Critical Care Deutschland GmbH, Hallbergmoos, Germany) dissolved in 100 % oxygen. Adequate anesthesia was monitored by testing the interclaw reflex. During CMR, heart rate was monitored. When necessary, additional anesthesia was provided by administering fentanyl (7.5µg every 30 minutes, Rotexmedica GmbH, Trittau, Germany) or ketamine (Ketaset 100 mg/ml, Zoetis, Berlin, Germany).

CMR was performed at day 6 after vessel injury using a whole-body 3.0 T Achieva X-series MR scanner (Philips Healthcare, Best, the Netherlands). *In vivo* CMR was performed according to previously established animal handling, anesthetic and CMR workflow protocols [6, 7]. For ^1^H measurements two flexible double array surface coils of 14 x 17 cm and 20 cm diameter (SENSE Flex M and SENSE Flex L surface coil, Philips Healthcare, Best, the Netherlands) were used. The CMR scan consisted of a ^1^H protocol including 3D time of flight (TOF) angiography, phase-contrast velocity encoded (VENC) measurements and high-resolution T2-weighted black blood sequences for vessel wall assessment.

The 3D time of flight (TOF) angiography of the carotid arteries was performed using a three-dimensional inflow (3DI) multichunk (MC) fast field echo (FFE) with water selective excitation (WATS; 3DI MC WATS). TOF angiography was performed using the following parameters: repetition time (TR) = 21.84 ms, echo time (TE) = 4.33 ms, flip angle (FA) = 16°. Furthermore, segmented gradient-echo phase contrast CMR (PC-CMR) was performed at the proximal, middle and distal part of the carotid artery (TR = 8.87 ms; TE = 5.22 ms; FA = 10°). The velocity encoding range was set at 150 cm/s in a through-plane direction. For vessel wall assessment a high-resolution T2-weighted black blood turbo spin echo sequences (TR = 1558 ms; TE = 60 ms; FA = 90°) was used. After acquisition of ^1^H reference scans, the pigs were removed from the magnet bore without losing the isocenter information, and the. ^19^F coil (7 × 12 cm dual tunable ^1^H/^19^F ellipsoidal coil (Philips Healthcare, Best, the Netherlands) was placed on the neck directly above the injured artery. The ^19^F ellipsoidal coil was tune and match for both ^19^F and ^1^H. ^1^H and ^19^F transmission could be switched by a small extra loop. Thereafter, pigs were repositioned into the scanner at the same isocenter position, which was confirmed by repeating ^1^H reference scans. ^19^F imaging was performed using a balanced steady state free precession (bSSFP) sequence centered at 58 ppm (3D acquisition, with a 3 x 3 x 3 mm³ isotropic voxel size; TR = 2.88 ms; TE = 1.00ms; FA = 30° ), as described in a previous applicability study [8]. Due to the frequency selective excitation at 58 ppm and the low isoflurane concentration applied (1.5 % v/v) no relevant ^19^F signals from isoflurane could be observed in the lung or adipose tissue.

After *in vivo* CMR scans were accomplished, pigs were sacrificed inside the scanner with potassium chloride and an overdose of pentobarbital (Narcoren, Boehringer Ingelheim, Ingelheim am Rhein, Germany). Heparin (10.000 I.E. Heparin-Natrium 25000, Ratiopharm GmbH, Ulm, Germany) was injected to prevent coagulation.

**Analysis of CMR datasets**

^1^H MR angiography of carotid arteries as well as ^19^F images and ^1^H/^19^F overlay were visualized using Horos^TM^ version 3.3.6 (Horos project, Annapolis, MD, USA). Carotid artery flow was assessed by Q flow MR images and automatically analysed using Circle CVI 42 version 5.11 (Circle Cardiovascular Imaging Inc., Calgary, AB, Canada).

For calculation of ^19^F Signal to noise ratio (SNR) *in vivo* all slices with ^19^F signal were included into the analysis. In every slice, SNR was calculated from the ratio of the mean of a region of interest (ROI) and the standard deviation of the noise of a ROI in a different slice located beside any tissue of the same data set. *In vivo* SNR was then calculated as the mean SNR of every slice included into the calculation.

For *ex vivo* assessment of ^19^F signal intensity, SNR was calculated for every cross-sectional image as the mean of a region of interest (ROI) and the standard deviation of the noise of a ROI in a different slice located beside any tissue of the same data set. Distance from bifurcation was obtained simultaneously and a profile of ^19^F SNR in dependence of slice location was plotted.

**Organ preparation and *ex vivo* scans**

Autopsy was performed and both carotid arteries and the carotid trunk were excised *in toto* and stored in 4% paraformaldehyde (PFA) for at least 7 days. To optimize image conditions, but using the same technical equipment and sequences, e*x vivo* scans were performed at 3.0 Tesla with optimized coil distance of only a few mm and an identical isotropic image resolution for ^1^H and ^19^F (1x1x1mm³) with an image acquisition duration of 19min. To precisely localize ^19^F signals in carotid arteries for a direct histological validation, high resolution images of total excised carotid arteries were carried out at 9.4 T using a Bruker AVANCEIII 400 MHz Wide Bore NMR spectrometer (Bruker, Rheinstetten, Germany). Here, ^19^F datasets were recorded using a 3D RARE sequence (RARE factor 32, TR 3.5 s, 330 x 450 µm in-plane resolution, Slice thickness 1 mm, 465 averages, Scan time 48 h). For exact anatomic localization, the ^19^F datasets were merged with corresponding 3D 1H RARE scans (RARE factor 16, TR 5 s, 120 x 120 µm in-plane resolution, Slice thickness 1 mm, 10 averages, Scan time 16 h).  The arteries were then cut in short axial slices with a medium thickness of 5 mm. Afterwards slices were embedded in paraffin. For further histological processing slices of 5 µm thickness were cut with a microtome (Jung Biocut 2035, Mikrotom, Leica Instruments GmbH, Nussloch, Germany).

**Histological protocols**

The H.E. Staining was carried out according to the following protocol:

Paraffin slices (5 µm) were incubated in a heat chamber at 60°C for 60 minutes. Slices were dewaxed in xylene (Carl Roth, Karlsruhe, Germany) and paraffin was removed using a series of ethanol with descending concentration. They were then incubation in hematoxylin solution (Merck KGaA, Darmstadt, Germany) for two minutes and rinsed in tap water followed by 10 seconds differentiation in 0.5% hydrochloric acid solution (PanReacAppliChem, Darmstadt, Germany). They were then rinsed in tap water again for one minute. Afterwards incubation in eosin solution (Carl Roth, Karlsruhe, Germany) was done for one minute. Slices were then subjected to a series of ethanol with ascending concentration and incubated in xylene. Cell count in H.E. stained sections was done semi-automatically using ImageJ [9].

Immunohistochemistry was carried out according to the following protocol:

Paraffin slices (5 µm) were incubated in a heat chamber at 60°C for 60 minutes. The slides with paraffin embedded sections were dewaxed with Roticlear® (Carl Roth, Karlsruhe, Germany) and rehydrated in a decreasing Ethanol row (100%, 96%, 70%, 50%). After a washing step in Aqua dest., a heat mediated antigen retrieval using Citrate buffer (pH 9, Thermo Fisher Scientific, Waltham, MA, USA) was performed. Slices were washed with cold tap water and then incubated in PBS for five minutes. Afterwards slices were incubated for 20 minutes in 20% hydrogen peroxide (Carl Roth, Karlsruhe, Germany). Slices were washed five minutes in PBS and incubated with anti-CD163 antibody ((clone 2A10/11, Bio-Rad Laboratories, Inc., Hercules, California, USA); or anti-CD14 antibody (dilution 1:100, clone MIL2, Bio-Rad Laboratories Inc., Hercules, CA, USA) for 60 minutes followed by a washing step in PBS. Afterwards slices were incubated with a biotinylated goat anti-mouse antibody for 30 minutes followed by another washing step in PBS. Then, slices were incubated with Horseradish peroxidase (HRP) conjugated Streptavidin for another 30 min. A ready-to-use aminoethyl carbazole (AEC) solution (Thermo Fisher Scientific) was used as a substratum with an incubation period of 8-10 min. Afterwards nuclear staining with hematoxylin (Gill II) for about 10 seconds was performed. Slices were washed in tap water, mounted with aqueous mounting medium (Aquatex, Merck Millipore, Burlington, Massachusetts, USA) and brightfield images were acquired using a DM4000M microscope (Leica, Wetzlar, Germany). Cell count in immunhistochemistry was done semi-automatically using ImageJ [9].

Immunofluorescence staining for differentiation of macrophage subpopulations

The slides with paraffin embedded sections were dewaxed with Roticlear® (Carl Roth, Karlsruhe, Germany) and rehydrated in a decreasing Ethanol row (100%, 96%, 70%, 50%). After a washing step in Aqua dest., a heat mediated antigen retrieval using Citrate buffer was performed. Unspecific binding was blocked with a blocking buffer (0.2% Fish Skin Gelatine, 0.5% BSA, 0.1% Saponin in PBS) for 1h. The tissue was washed in PBS and incubated by the anti-CD163 antibody (clone EDHu-1, 7.5µg/ml, #NB110-40686, Novus biological, Minneapolis, USA) over night. A secondary anti-mouse-Alexa Fluor 660 (#A-21055, 2µg/ml, ThermoFisher Scientific, Waltham, USA) was used for 1h. After additional washing steps, the sections were incubated with anti-CD68 antibody (ab125212, 7.5µg/ml, abcam, Cambridge, UK) antibody for 10h. A secondary anti-rabbit-Alexa Fluor 594 (A-21207, 2µg/ml, ThermoFisher Scientific, Waltham, USA) was used for 1h. The sections were washed in PBS and unwanted autofluorescence was diminish using the Vector® TrueVIEW® Autofluorescence Quenching Kit (Vector Laboratories, Burlingame, USA) for 2min. The sections were mounted with Prolong Diamond with DAPI (ThermoFisher Scientific, Waltham, USA) and images were acquired using a fluorescence microscope (DM6B, Leica, Wetzlar, Germany).

**Blood sampling**

At day 0, 3 and 6 venous blood samples were taken in one EDTA and two heparin tubes. Basic blood cell count was performed from EDTA tubes.

**Table 1**

| **Grade 1**  **Grade 2**  **Grade 3** | **Tachypnoe**  **>20/min**  **>25/min**  **>30/min** | **Rushing**  **During infusion**  **Lasting <1 h**  **Lasting >1 h** | **Tachycardia**  **>100/min**  **>120/min**  **>140/min** | **Abnormal Behavior** |
| --- | --- | --- | --- | --- |
| **Grade 1** | **2/8** | **1/8** | **2/8** | **0/8** |
| **Grade 2** | **1/8** | **0/8** | **2/8** | **0/8** |
| **Grade 3** | **0/8** | **0/8** | **0/8** | **0/8** |

**Table I:** **Reactions to infusion of perfluorooctyl bromide nanoemulsion**

Eight pigs received the body weight-adapted infusion of the perfluorooctyl bromide nanoemulsion (4 with BA and 4 with BA + ECDN). Shown are the numbers of animals with the respective reaction.

**Table 2**

|  | **Day 0** | **Day 3**  **(Before PFOB-NE)** | **Day 3**  **(After PFOB-NE)** | **Day 6** | **P-value** |
| --- | --- | --- | --- | --- | --- |
| **Leukocytes (x10^3^/µl)** | 8.2 ± 2.6 | 8.3 ± 1.5 | 8.5 ± 3.8 | 11.2 ± 2.2 | 0.2010 |
| **Hemoglobin (g/dl)** | 11.2 ± 2.1 | 12.2 ± 2.6 | 10.3 ± 1.3 | 11.5 ± 2.5 | 0.5625 |

**Table II:** **Circulating leukocytes and hemoglobin after vascular injury**

Shown are white blood cell count (leukocytes) and hemoglobin (Hb) of all pigs after vascular injury. Analyzed time points were baseline, 3 days (before and after application of PFOB-NE) and 6 days after vascular injury. Shown are mean values ± standard deviation. *P*-values were calculated by One-Way ANOVA for repeated measures and Bonferroni post-hoc tests.

**Literature**

1. Granada, J.F., K. Milewski, H. Zhao, J.J. Stankus, A. Tellez, M.S. Aboodi, G.L. Kaluza, C.G. Krueger, R. Virmani, L.B. Schwartz, and A. Nikanorov, *Vascular response to zotarolimus-coated balloons in injured superficial femoral arteries of the familial hypercholesterolemic Swine.* Circ Cardiovasc Interv, 2011. **4**(5): p. 447-55.

2. Ishii, A., F. Vinuela, Y. Murayama, I. Yuki, Y.L. Nien, D.T. Yeh, and H.V. Vinters, *Swine model of carotid artery atherosclerosis: Experimental induction by surgical partial ligation and dietary hypercholesterolemia.* American Journal of Neuroradiology, 2006. **27**(9): p. 1893-1899.

3. Busnelli, M., S. Manzini, A. Froio, A. Vargiolu, M.G. Cerrito, R.T. Smolenski, M. Giunti, A. Cinti, A. Zannoni, B.E. Leone, M. Forni, M.L. Bacci, G.M. Biasi, R. Giovannoni, and M. Lavitrano, *Diet induced mild hypercholesterolemia in pigs: local and systemic inflammation, effects on vascular injury - rescue by high-dose statin treatment.* PLoS One, 2013. **8**(11): p. e80588.

4. Busnelli, M., A. Froio, M.L. Bacci, M. Giunti, M.G. Cerrito, R. Giovannoni, M. Forni, F. Gentilini, A. Scagliarini, G. Deleo, C. Benatti, B.E. Leone, G.M. Biasi, and M. Lavitrano, *Pathogenetic role of hypercholesterolemia in a novel preclinical model of vascular injury in pigs.* Atherosclerosis, 2009. **207**(2): p. 384-90.

5. Flogel, U., Z. Ding, H. Hardung, S. Jander, G. Reichmann, C. Jacoby, R. Schubert, and J. Schrader, *In vivo monitoring of inflammation after cardiac and cerebral ischemia by fluorine magnetic resonance imaging.* Circulation, 2008. **118**(2): p. 140-8.

6. Bonner, F., M.W. Merx, K. Klingel, P. Begovatz, U. Flogel, M. Sager, S. Temme, C. Jacoby, M. Salehi Ravesh, C. Grapentin, R. Schubert, J. Bunke, M. Roden, M. Kelm, and J. Schrader, *Monocyte imaging after myocardial infarction with 19F MRI at 3 T: a pilot study in explanted porcine hearts.* Eur Heart J Cardiovasc Imaging, 2015. **16**(6): p. 612-20.

7. Jacoby, C., S. Temme, F. Mayenfels, N. Benoit, M.P. Krafft, R. Schubert, J. Schrader, and U. Flogel, *Probing different perfluorocarbons for in vivo inflammation imaging by 19F MRI: image reconstruction, biological half-lives and sensitivity.* NMR Biomed, 2014. **27**(3): p. 261-71.

8. Rothe, M., A. Jahn, K. Weiss, J.H. Hwang, J. Szendroedi, M. Kelm, J. Schrader, M. Roden, U. Flogel, and F. Bonner, *In vivo (19)F MR inflammation imaging after myocardial infarction in a large animal model at 3 T.* MAGMA, 2019. **32**(1): p. 5-13.

9. Schneider, C.A., W.S. Rasband, and K.W. Eliceiri, *NIH Image to ImageJ: 25 years of image analysis.* Nat Methods, 2012. **9**(7): p. 671-5.
